# Single flap approach combined with periosteal pedicle flap as a regenerative procedure: A case report

**DOI:** 10.1002/cap.70052

**Published:** 2026-02-19

**Authors:** Mattia Severi, Massimo Viola, Leonardo Trombelli

**Affiliations:** ^1^ Research Centre for the Study of Periodontal and Peri‐Implant Diseases University of Ferrara Ferrara Italy; ^2^ Operative Unity of Dentistry of Ferrara Ferrara Italy; ^3^ Private Practice Rome Italy

**Keywords:** heterografts, periodontitis, periosteum, regeneration, wound healing

## Abstract

**Background:**

Single flap approach (SFA) is a validated procedure for obtaining clinical attachment gain and pocket closure when applied to deep intraosseous defects. However, the presence of an interdental crater as well as the tooth location were associated with a lower rate of primary intention closure, possibly jeopardizing the outcome of the procedure. In order to favor primary closure, the use a periosteum pedicle flap (PPF) was proposed. The present case report showed the effect of a combination of SFA with PPF for the treatment of a severe, isolated intrabony defect at a molar site associated with a deep interproximal crater.

**Methods:**

A patient presenting two 6‐mm deep bleeding periodontal pockets, in association with an intrabony defect, at the mesio‐buccal and mesio‐lingual aspect of #19 was treated with the combination of SFA and PPF. Intrabony component of the defect was filled with a combination of deproteinized bovine bone mineral mixed with a gel composed by polynucleotides and hyaluronic acid.

**Results:**

A dehiscence of the primary flap was observed at the interproximal area. Primary closure was still present due to the presence of the underlying periosteal pedicle flap. At the 12‐months follow up, pocket closure was observed at both mesio‐buccal and mesio‐lingual aspects. The radiographical examination showed a substantial remineralization of the infrabony component

**Conclusion:**

The combination of SFA with PPF may be a promising approach for the treatment of intrabony defects associated with unfavorable conditions of soft tissues located at molar sites.

**Key points:**

- **Why is this case new information?**
- The present case reports that single flap approach may be successfully combined with a periosteal pedicle flap to correct intrabony defects associated with unfavorable conditions of soft tissues located at molar sites.
- **What are the keys to successful management of this case?**
- Careful dissection of the flap to isolate and maintain the integrity pf the periosteal pedicle flap
- Complete debridement of both the intrabony defects and of the root
- Stabilization of the periosteal pedicle flap above the defect in order to contribute to the primary intention healing
- **What are the primary limitations to success in this case?**
- Absence of unfavorable anatomical conditions possibly jeopardizing the primary closure of the surgical wound
- Proximity with mental nerve due to possible lesions during the partial thickness dissection

**Plain language summary:**

The present paper explored the use of a surgical technique combining a buccal single flap elevation and a periosteal pedicle to treat a periodontal pocket associated with a deep intrabony defect. A buccal split‐thickness incision was performed to both visualize the osseous defect and isolate a periosteal pedicle. The defect was debrided and filled with a combination of a xenograft and gel composed of polynucleotides and hyaluronic acid. The periosteal pedicle was rotated and sutured above the filled defect. The primary split‐thickness flap was sutured, covering both the defect and periosteal pedicle. Twelve months after surgery, no periodontal pocket was present. At the radiographic examination, substantial remineralization of the defect was observed.

## INTRODUCTION

Single flap approach (SFA)^1^ is one of the most validated procedures for obtaining a relevant clinical attachment gain and pocket closure when applied to deep intraosseous defects.^2^, ^3^ SFA was shown to effectively combine significantly greater improvement of clinical parameters with limited postoperative discomfort when compared to papilla preservation techniques.^4^, ^5^ This improvement is at least in part associated with better condition of primary intention, secluded closure of the interdental tissue.^6^ In this respect, several factors such as the presence of an interdental crater as well as the tooth location (anterior vs. posterior) were associated with a lower rate of primary intention closure, possibly jeopardizing the outcome of the regenerative procedure.^7^ Moreover, the combined use of a resorbable membrane and a bone substitute with SFA was shown to increase the proportion of defects showing an incomplete wound closure, possibly due to a reduction of the revascularization of the surgical area.^7^, ^8^

In order to enhance the flap revascularization following a regenerative surgery at intrabony defects, thus favoring wound stability and primary closure conditions, the use a periosteum pedicle flap (PPF) was previously proposed.^9^ Briefly, the technique consisted in the elevation of a buccal partial thickness flap, in order to isolate the PPF, combined with a lingual/palatal full thickness flap. After defect debridement, the PPF was rotated to cover the interproximal defect without suturing it. The buccal partial thickness flap was then repositioned to achieve primary intention closure. The use of PPF, either alone or with a mixture of hydroxyapatite (HA) and beta‐tricalcium phosphate (b‐TCP), for the treatment of either 2 or 3‐walls intrabony defects resulted in significant probing depth (PD) reduction and clinical attachment level (CAL) gain.^10^, ^11^ Moreover, histological evidence of novel periodontal attachment formation associated with the use of PPF was observed.^10^

The present case report showed the effect of a combination of SFA with PPF for the treatment of a severe, isolated intrabony defect at a molar site associated with a deep interproximal crater. This combination was proposed in order to enhance the condition of wound stability and primary intention closure at a site with challenging defect configuration and soft tissue conditions.

## MATERIALS AND METHODS

In October 2022, a 57‐years‐old, non‐smoker, systemically healthy woman was treated at the operative unit of dentistry of Ferrara for a stage III grade C generalized periodontitis.^12^ Periodontal re‐evaluation was performed 8 weeks after the completion of stage I and II.^13^ Two residual bleeding pockets of 12 and 10 mm, respectively, were still present at the mesial aspect of tooh #19, in association with a severe intrabony defect. A periodontal regenerative procedure consisting in a modified papilla preservation flap^14^ in combination with a deproteinized bovine bone mineral (DBBM) graft and a native collagen membrane was used to correct the intrabony component of the defect as well as to close the residual pockets. However, a complete necrosis of the interproximal tissue in association to a second intention healing phase leading to membrane/graft exposure was observed at the 2 weeks postoperatively.

**FIGURE 1:**
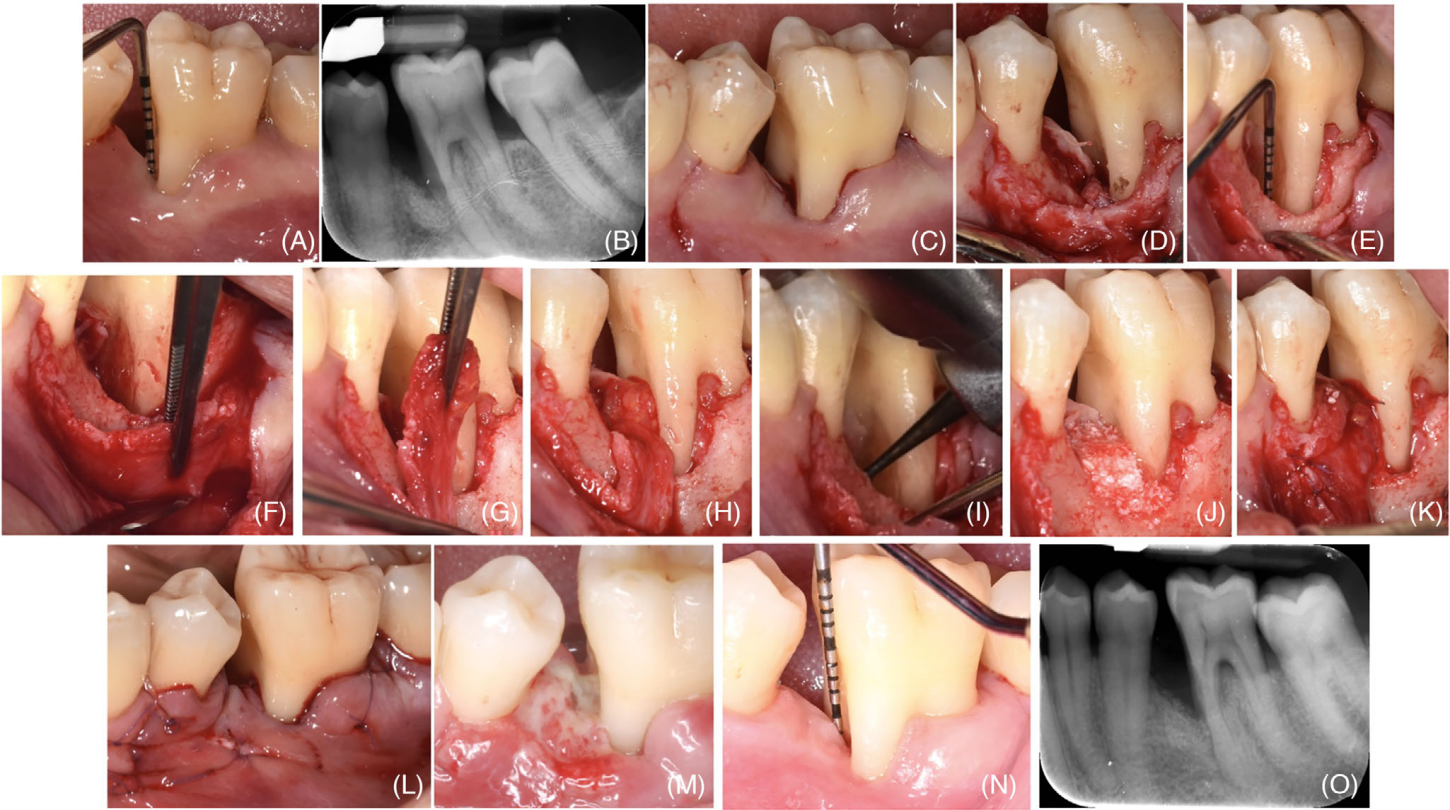
(A, B) Clinical and radiographical aspect of tooth #19. (C, D) Partial thickness dissection was performed to isolate a primary buccal flap leaving periosteum lining the bone crest. (E) Combined 2‐ and 3‐walls defect at the end of debridement. (F‐H) Periosteum was incised distally and apically in order to mobilize a pedicle. (I) Cortical perforations were performed. (J) Intrabony component of the defect was filled with a combination of deproteinized bovine bone mineral (DBBM) and a gel composed of polynucleotides and hyaluronic acid. (K, L) Periosteal pedicle was sutured to the firm lingual papilla and, subsequently, primary flap was sutured obtaining primary closure. (M) Necrosis of the interdental primary flap. Primary closure was provided by the underlying periosteal pedicle flap. (N, O) At the 12‐months follow up a 3 mm probing depth (PD) was presented. Radiographical examination showed a substantial remineralization of the defect.

Eighteen months after surgery, despite the enrollment in a stringent supportive periodontal care with a frequency of 3‐month session, two 6‐mm deep bleeding residual periodontal pockets, in association with an intrabony defect, were still present at the mesio‐buccal and mesio‐lingual aspect of #19 (Figure 1A,B).

Therefore, a surgical regenerative re‐entry was planned to both correct the residual intrabony component of the defect and to close the residual pocket. The patient provided a written informed consent prior to surgical treatment. All clinical procedures have been performed in accordance with the Declaration of Helsinki and the Good Clinical Practice guidelines (GCPs). ^15^Surgery was performed by one experienced periodontal surgeon (MS) using 2.5 magnifying loops.

Local anesthesia was attained using articaine with 1:100,000 epinephrine administered by local infiltration.

A buccal vertical releasing incision was performed mesial to #20 and prolonged intrasulcularly from #20 to #18. Interproximal tissue between #20 and # 19 was incised according to SFA principles.^1^, ^6^ Then, a sharp dissection using a 15C blade was performed starting from the vertical incision, aimed at raising a partial thickness gingival primary flap, taking care in leaving a periosteal layer lining the buccal bone crest. (Figure 1C,D). In order to access the intrabony defect, the periosteal layer was elevated from the bone crest using a microsurgical periosteal elevator (PTROM, Hu‐Friedy) and tunnelling knives with varying angulated sharp edges (KPAX, TKN1X and TKN2X, Hu Friedy). No elevation of both interdental papilla and lingual gingival tissue was performed.

Root debridement was performed using ultrasonic device with periodontal inserts (AIRFLOW Prophylaxis Master, EMS). Granulation tissue as well as biomaterial remnants from the previously performed surgery were carefully removed from the intrabony component of the defect.

At the end of debridement, defect morphology consisted of combined 2 and 3‐walls components from its coronal portion to the bottom, respectively (Figure 1E). The periosteal layer was then incised at its most apical and distal portion in order to design a periosteal pedicle flap^9^ that could be mesially rotated (Figure 1F–H). Cortical perforations were performed at the interproximal bony wall of the defect to increase vascular supply and osteogenic cell source^16^ (Figure 1I).

A particulate DBBM (Bio‐Oss, size 0.25–1 mm; Geistlich Pharma AG) graft mixed with a gel composed by polynucleotides and hyaluronic acid (Rengenfast, Mastelli Srl, San Remo, Italy) was used to fill the intrabony component of the defect up to its coronal portion (Figure 1J).

The periosteal pedicle flap was sutured to the lingual papilla by mean of an internal mattress suture using a resorbable 6/0 suture in order to cover the buccal and coronal portion of the defect. Subsequently, single interrupted sutures were used to stabilize the periosteal pedicle flap mesially to the soft tissues still attached to the bone crest due to partial thickness elevation. Finally, the gingival flap was sutured, using interproximal modified internal mattress sutures, to obtain primary intention closure. Vertical releasing incision was sutured using single interrupted sutures (Figure 1K,L)

The patient was instructed to avoid any compression of the surgical site and not to chew or brush in the treated area for 2 weeks. A 0.12% chlorhexidine mouthrinse (Curasept ADS Trattamento Rigenerante; Curaden Healthcare)* was prescribed (1‐minute rinse b.i.d. for 3 weeks). Sutures were removed at 2 weeks post‐surgery.

## RESULTS

A dehiscence of the primary flap was observed at the interproximal area. However, a primary closure was still present due to the presence of the underlying periosteal pedicle flap. No evidence of exfoliation of biomaterial was observed (Figure 1M).

Patient was included in a personalized program of periodontal supportive therapy consisting in three‐monthly recall frequency based on Periorisk score.^17^, ^18^

At the 12‐months follow up, pocket closure was observed at both mesio‐buccal and mesio‐lingual aspects. The radiographical examination showed a substantial remineralization of the infrabony component (Figure 1 N,O).

## DISCUSSION

The present case report suggests that the use of single flap approach in combination with a periosteal pedicle flap may be a suitable option for treating intrabony defects associated with conditions that may jeopardize primary intention closure such as the presence of an interproximal soft tissue crater at a molar site.

SFA, either alone or in association with different combination of regenerative devices, is a validated and efficacious approach for the treatment of intrabony defects.^3^, ^4^, ^8^, ^19^ The preservation of an intact interdental papilla facilitates flap repositioning and suturing, thus optimizing primary intention wound closure and the re‐establishment the interdental vascular supply. However, patient and tooth‐related factors, such as an unfavorable anatomy of interdental soft tissue, the presence of a wide 2–3 wall defect at a molar site and the use of bone substitutes may negatively affect the rate of primary intention closure and, consequently, affect the predictability of the regenerative outcome.^7^, ^20^ Finally, defect anatomy plays an important role in periodontal‐regenerative procedures of intrabony defects. It is well known that the number of residual bony walls (1‐, 2‐or 3‐wall defects), the depth of the intrabony component as well as the width of the angle between the bony wall of the defect and the long axis of the tooth may reduce the architectural support, revascularization potential, cellular recruitment potential, and clot stability.^21^, ^22^, ^23^, ^24^ An overlap of these conditions may increase the need for combined approaches aimed at enhancing wound stability in order to achieve predictable periodontal regeneration.^25^, ^26^

In the present case report, periosteum pedicle flap was used for: (i) its osteogenic and angiogenic properties, and (ii) increasing the chance of primary intention closure in presence of adverse anatomical condition of the interproximal tissue above the intrabony defect.^20^ The use of SFA provided a firm lingual papilla that could be used to suture and stabilize both periosteal pedicle flap and the primary gingival flap. Periosteal pedicle flap was used due to the biological properties of periosteum as well as to provide a second layer for primary intention closure that could possibly compensate a failure of the primary gingival flap closure.

The capacity of the surgical manipulation of the periosteum to induce novel bone formation was previously showed in several studies.^27^, ^28^ The use of periosteum as an osteogenic, vascularized membrane was shown to be clinically effective in reconstructing both periodontal intrabony defects^10^, ^11^ and peri‐implant bone dehiscences.^29^, ^30^ Moreover, evidence of novel periodontal attachment as well as of newly formed peri‐implant buccal bone were observed in histological human studies in both intraosseous^10^ and peri‐implant bone defects.^31^

The use of periosteum was extensively proposed in order to enhance wound stability and primary intention closure in guided bone regeneration (GBR) procedures with both resorbable^32^, ^33^ and non‐resorbable^34^ membranes. Finally, the use of a PPF rather than a resorbable/non‐resorbable membrane may have had increased the vascularization of the wound, thus avoiding the exposure and consequent exfoliation of the bone graft material, as previously reported in studies where SFA was used in in combination with a resorbable membrane and a bone substitute.^7^, ^8^

In the present report, the combination of SFA with a PPF acted as a double layer surgical wound. PPF sutured to the lingual, untouched interproximal tissue completely covered the grafted defect, thus bringing vascular supply and providing wound stability. Although a partial necrosis of the mucosal portion of the flap was observed at 2 weeks, the presence of the underlying periosteal flap allows for maintaining a primary intention closure with no exfoliation of the graft material being observed. Apart from its osteogenic properties, this observation seems to suggest that the periosteal layer may have acted as a “biologic membrane” to enhance wound stability conditions. In this respect, preclinical animal study^35^ evaluated new bone formation in critical defects created in the rat frontal bone covered with either a non‐resorbable membrane (control group) or a periosteal pedicle flap (test group). At the 12‐weeks histological analysis no differences in the amount of novel bone formation were observed as well as in the rate of completely reconstructed defects between test and control group.^35^ Overall, within the limitations of the present report, it can be concluded that the combination of SFA with PPF may be a promising approach for the treatment of intrabony defects associated with unfavorable conditions of soft tissues located at molar sites. However, due to the explorative nature of the present case report, it is difficult to isolate the single impact of the PPF with respect to several variables included in the surgical procedure, such as the use of DBBM in combination with gel composed by polynucleotides and hyaluronic acid as well as cortical perforations. Further studies are needed to evaluate the contribution of each of these factors to clinical results of the procedure.

## CONCLUSION

The present proof‐of‐principle case report indicates that single flap approach in combination with a periosteal pedicle flap could be a feasible therapeutic option in cases where anatomical conditions may jeopardize primary intention healing. Specifically, the isolation of a periosteal pedicle could be easily performed by means of a partial thickness flap and biological features of periosteum may facilitate healing dynamic due to its osteo‐ and angiogenic properties.

Whether and to what extent the combination of single flap approach and periosteal pedicle flap could be more effective than single flap approach alone should be investigated in further longitudinal studies.

## AUTHOR CONTRIBUTIONS

**Mattia Severi**: Conceptualization (lead); investigation (lead); writing—original draft preparation (lead); writing—review & editing (equal). **Massimo Viola**: Writing—original draft preparation (supporting). **Leonardo Trombelli**: Writing—review & editing (equal).

## CONFLICT OF INTEREST STATEMENT

Mattia Severi and Massimo Viola declare no conflicts of interest. Leonardo Trombelli received grants and fees for lectures and courses by Geistlich Pharma AG, Wolhusen, Switzerland.

## Data Availability

Data sharing is not applicable to this article as no datasets were generated or analyzed during the current study.
